# Sexual Orientation, Sexual Arousal, and Finger Length Ratios in Women

**DOI:** 10.1007/s10508-021-02095-5

**Published:** 2021-07-23

**Authors:** Luke Holmes, Tuesday M. Watts-Overall, Erlend Slettevold, Dragos C. Gruia, Jamie Raines, Gerulf Rieger

**Affiliations:** 1 Department of Psychology, University of Essex, Colchester, CO4 3SQ UK; 2 Present Address: School of Psychology, University of East London, London, UK; 3 Present Address: Department of Medicine and Health Sciences, University of East Anglia, Norwich, UK

**Keywords:** Sexual orientation, Sexual arousal, Prenatal influences, Masculinity–femininity

## Abstract

In general, women show physiological sexual arousal to both sexes. However, compared with heterosexual women, homosexual women are more aroused to their preferred sex, a pattern typically found in men. We hypothesized that homosexual women’s male-typical arousal is due to their sex-atypical masculinization during prenatal development. We measured the sexual responses of 199 women (including 67 homosexual women) via their genital arousal and pupil dilation to female and male sexual stimuli. Our main marker of masculinization was the ratio of the index to ring finger, which we expected to be lower (a masculine pattern) in homosexual women due to increased levels of prenatal androgens. We further measured observer- and self-ratings of psychological masculinity–femininity as possible proxies of prenatal androgenization. Homosexual women responded more strongly to female stimuli than male stimuli and therefore had more male-typical sexual responses than heterosexual women. However, they did not have more male-typical digit ratios, even though this difference became stronger if analyses were restricted to white participants. Still, variation in women's digit ratios did not account for the link between their sexual orientation and their male-typical sexual responses. Furthermore, homosexual women reported and displayed more masculinity than heterosexual women, but their masculinity was not associated with their male-typical sexual arousal. Thus, women’s sexual and behavioral traits, and potential anatomical traits, are possibly masculinized at different stages of gestation.

## Introduction

Women show, on average, substantial physiological sexual arousal to sexual stimuli featuring either females or males, regardless of their self-reported sexual orientation. Conversely, most men show substantial sexual arousal to their preferred sex but not to their non-preferred sex (Bailey, 2009). This sex difference appears to be robust, as it has been reported with several measures of sexual response including genital arousal (Chivers et al., 2004; Rieger et al., 2015, 2016; Suschinsky et al., 2009), pupil dilation (Attard-Johnson et al., 2016; Rieger & Savin-Williams, 2012; Rieger et al., 2015), viewing time (Ebsworth & Lalumière, 2012; Israel & Strassberg, 2009), and neural responses (Safron et al., 2019; Sylva et al., 2013). Substantial sexual arousal to both female and male sexual stimuli can therefore be described as female-typical and substantial sexual arousal only to the preferred sex as male-typical (Chivers et al., 2007).

As aforementioned, this sex difference in sexual arousal has been well established (Chivers et al., 2004; Holmes et al., 2021; Rieger et al., 2015; Watts et al., 2018c). Yet, this sex difference only applies on average and there are exceptions. One of these exceptions involves homosexual women. Although, like heterosexual women, homosexual women show arousal to both sexes, they also have greater sexual responses to their preferred sexual stimuli (females) compared to their non-preferred sex (males); this is the case when measuring their genital arousal (Chivers et al., 2004, 2007; Rieger et al., 2016), pupil dilation (Rieger & Savin-Williams, 2012; Rieger et al., 2015), viewing time (Ebsworth & Lalumière, 2012; Lippa, 2012), and neural responses (Safron et al., 2017). Thus, the arousal patterns of homosexual women can be described as “male-typical,” as stronger responses to one’s preferred sex are usually found in men (Bailey, 2009). We stress, however, that while homosexual women show a preference for one sex, they still respond, on average, to stimuli featuring either sex. As such, their response patterns can only be considered male-typical in comparison to those of heterosexual women, and we predicted such a pattern in the present research (Hypothesis 1). Bisexual women are, on average, between heterosexual and homosexual women in their sexual responses, showing more of a preference for female stimuli than heterosexual women, but less of a preference than homosexual women (Rieger et al., 2016; Timmers et al., 2015).

It is possible that the arousal patterns of homosexual women are influenced by factors that typically influence sexual arousal in men. A prominent candidate is exposure to androgens during gestation. In mammals, exposure to prenatal androgens—specifically testosterone—accounts for the majority of sex differences in brain and behavior (Breedlove, 2017; Morris et al., 2004). In humans, our knowledge of the effects of prenatal androgen exposure is informed by genetic conditions which impact the availability of androgens or the individual’s sensitivity to them (Hines, 2009). One such condition is congenital adrenal hyperplasia (CAH), which causes excessive production of androgens from gestation onwards. During their childhood, these genetic females are more likely to engage in male-typical play (Pasterski et al., 2005), and in adulthood, they are more likely to express bisexual or homosexual attractions (Hines et al., 2004; Meyer-Bahlburg et al., 2008; Zucker et al., 1996). Another relevant condition is complete androgen insensitivity syndrome (CAIS), which is an immunity to the effects of androgens at all stages of development. Genetic males with CAIS typically report sexual orientations toward males (Wisniewski et al., 2000) and show neural responses to both male and female sexual stimuli, and therefore have female-typical sexual responses (Hamann et al., 2014). Thus, levels of early androgen exposure may affect not only the development of general sex differences, but also the formation of sexual orientation and sexual arousal patterns within each sex (Bailey et al., 2016; Breedlove, 2010).

It is difficult to measure the level of androgens a human fetus is exposed to in utero, and research on the subject is often informed by postnatal biomarkers assumed to reflect prenatal androgen exposure. Of these, the most researched is the ratio of the length of the second digit to that of the fourth digit (2D:4D). There is a robust sex difference in this ratio; with men having a lower 2D:4D than women (Grimbos et al., 2010; Xu & Zheng, 2015). This sex difference emerges early in fetal development (Galis et al., 2010). Furthermore, females with CAH have lower (more male-typical) 2D:4D in the right hand than females without CAH (Brown et al., 2002a, 2002b; Ciumas et al., 2008; Ökten et al., 2002), whereas genetic males with CAIS have 2D:4D comparable to those of typically developed females (Berenbaum et al., 2009). Thus, these digit ratios may indeed reflect exposure to androgens during prenatal development.

Sexual orientation in women has repeatedly been linked to 2D:4D, with homosexual women having lower (more male-typical) 2D:4D than heterosexual women on average (Kraemer et al., 2006; Putz et al., 2004; Rahman, 2005; Wallien et al., 2008; Watts et al., 2018a), and we predicted the same pattern in present research (Hypothesis 2). A meta-analysis of 34 independent samples totaling 5,828 participants confirmed that women with a non-heterosexual orientation had lower 2D:4D in the left and right hands, compared with heterosexual women, Hedge’s *g’s* = 0.23 and 0.29, 0.04 < 95% CIs < 0.51 (Grimbos et al., 2010). The evidence for a link between women’s sexual orientation and 2D:4D has not been consistent across hands, with some studies finding a relationship only in the right hand and not in the left (Swift-Gallant et al., 2020). However, the aforementioned meta-analysis found an association between women’s 2D:4D and sexual orientation in both hands (Grimbos et al., 2010), and we found an association between 2D:4D and sexual orientation in the left hand in a previous project (Watts et al., 2018a). Thus, in the present research, we performed all analyses on both hands. Unlike in women, variations in 2D:4D are not consistently linked to differences in sexual orientation in men, and digit ratios may only serve as a reliable biomarker of early androgen exposure with respect to the development of female sexual orientation (Swift-Gallant et al., 2020).

If homosexual women were indeed exposed to elevated levels of prenatal androgens compared with heterosexual women, as reflected in their male-typical digit ratios, it could mean that prenatal androgenization influenced both their sexual orientation toward women and their male-typical arousal patterns. For this reason, the putative marker of prenatal androgen exposure, their 2D:4D, could account for the relationship between women’s sexual orientation and their sexual arousal patterns. Statistically this implies that 2D:4D would mediate the relationship of women’s sexual orientation with their sexual arousal patterns (Hypothesis 3).

A different line of research links sexual orientation to masculinity and femininity, which can be defined as opposite poles of an encompassing psychological and behavioral trait (Lippa, 1991, 2005, 2008). Homosexual women are more masculine (and less feminine) than heterosexual women in their self-reported behaviors and interests (Lippa, 2005). Furthermore, others perceive homosexual women as more masculine than heterosexual women, based on the way they appear, sound, or move; and this observable difference emerges in their early childhood (Johnson et al., 2007; Rieger et al., 2010; Watts et al., 2018b). We predicted such differences in masculinity–femininity, dependent on women's sexual orientation, in the present work (Hypothesis 4).

These non-sexual psychological and behavioral traits of homosexual women could also be indicators of their masculinization by hormone exposure during the prenatal period (Bailey et al., 2016; Bailey & Zucker, 1995; Brown et al., 2002a, 2002b; Swift-Gallant et al., 2020). Again, as with their sexual arousal, we must stress that they are only “masculinized” compared to other women, and not in comparison with biological males. Measures of masculinity–femininity could therefore be used as a proxy of prenatal androgenization to explain their male-typical sexual arousal patterns. This hypothesis has been tested by assessing women’s non-sexual traits via self-reported and observer-rated masculinity–femininity, and their sexual arousal via the measure of genital response and pupil dilation to female and male sexual stimuli. Homosexual women were more masculine in their observed-rated non-sexual behaviors, as well as their own self-reports, and had more male-typical sexual responses than heterosexual women. Yet, these two findings were not interlinked; women’s non-sexual masculinity did not mediate the relationship of sexual orientation with sexual arousal (Rieger et al., 2016). Perhaps male-typical behaviors and sexual arousal patterns in women develop independently of each other. Yet, there is also the possibility that a null finding was obtained by chance. Thus, in addition to testing whether women’s sexual responses were related to their digit ratios, we re-examined whether behavioral masculinity of homosexual women could explain their male-typical sexual arousal patterns (Hypothesis 5).

Similar to their genital arousal patterns, bisexual women are intermediate between heterosexual and homosexual women in their masculinity–femininity (Lippa, 2005, 2008). Perhaps they are also intermediate with respect to their 2D:4D, although, to our knowledge, this has not been explicitly studied previously.

The present research aimed to assess the relationship between sexual orientation, sexual arousal patterns, and proxies of prenatal androgen exposure. Sexual orientation was assessed via self-report, sexual arousal via genital blood flow and pupil dilation, and the proxies of prenatal androgen exposure employed were finger length ratios (2D:4D), self-reported adulthood and childhood masculinity–femininity, and observer-rated masculinity-femininity based on short video interview clips of participants.

Based on the reviewed literature, the following hypotheses were tested:

1. Homosexual women are more male-typical than heterosexual women in their sexual arousal, with stronger arousal to their preferred sex than their non-preferred sex. In comparison, heterosexual women show similar arousal to both sexes, and bisexual women are intermediate between the two groups in their arousal patterns.
2. Homosexual women have, on average, more male-typical (lower) 2D:4D than heterosexual women. We speculate that bisexual women will be intermediate in their 2D:4D.
3. The relationship between sexual orientation and male-typical sexual arousal in women is mediated by a putative marker of androgen exposure, 2D:4D.
4. Homosexual women are, on average, more masculine than heterosexual women in their non-sexual self-concepts and behaviors, and bisexual women are intermediate between the two groups.
5. The relationship between sexual orientation and male-typical sexual arousal in women is mediated by their masculine self-concepts and behaviors.

## Method

### Participants

#### Target Participants

Our sample size was planned based on previous studies that used methodologies identical to ours. These studies computed correlations between female sexual orientation and either genital arousal or pupil dilation to male or female sexual stimuli, or computed a correlation between female sexual orientation and 2D:4D measures. The reported correlations were 0.20, 0.27 and 0.30, respectively (Rieger et al., 2016; Watts et al., 2018a). A power analysis conducted in G*Power determined that a sample size of 195 would be necessary for the smallest estimated effect (*r* = 0.20) to achieve significant results with a power of 0.80. A total of 199 women were recruited via Pride festivals, online news sites for lesbian women, and university mailing lists. Using a 7-point scale (Kinsey et al., 1953), women self-identified as “exclusively straight” (*n* = 44), “mostly straight” (*n* = 42), “bisexual leaning straight” (*n* = 15), “bisexual” (*n* = 18), “bisexual leaning lesbian” (*n* = 13), “mostly lesbian” (*n* = 26), or “exclusively lesbian” (*n* = 41). The mean (SD) age of the sample was 24.22 (6.98), and most were Caucasian (78%), followed by Chinese (5%), Black (4%), and other ethnicities. Eighteen of the 199 participants in the present study were identical twins whose data have been previously published (Watts et al., 2018a, 2018c). Since identical twins are comparable to non-twins in their sexual arousal patterns, and in the interest of maximizing statistical power in the present sample, we decided to include these individuals in the analyses. 169 of the 199 participants have been reported on previously in a publication on sex differences in arousal and 2D:4D (Holmes et al., 2021).

Due to some participants opting out of the genital arousal component, others not responding to our messages to provide additional data (some participants were invited to provide 2D:4D data only after their original visit to the laboratory), and pupil data loss because of problems with the apparatus, genital arousal data were available for 184 women, pupil dilation data for 175 women, and 2D:4D data for 182 women. Computations of multiple imputations (5 total) were conducted across all examined variables using linear regression analyses as the model type. The imputed data suggested that if below analyses were repeated with missing data being imputed, in order to have an entire set of 199 data points across all variables, it changed neither the direction, nor magnitude, nor significance of effects. Thus, we decided to focus on analyses with observed data, even if that meant that participant numbers varied across analyses. The exact number of participants included in each stage of analysis can be found in the captions of the corresponding tables and figures, and a full listing is given in Table 1.

**Table 1:** Means, confidence intervals, standard deviations, and sample sizes for variables, split by sexual orientation groups

	Genital arousal to females over males	Pupil dilation to females over males	Right-hand 2D:4D	Left-hand 2D:4D	Self-reported childhood masculinity	Self-reported adulthood masculinity	Observer-rated adulthood masculinity
Heterosexual (Kinsey 0–1)	.07 [− .09, .22] (.72, *N* = 82)	.15 [− .01, .30] (.61, *N* = 64)	.975 [.965, .985] (.044, *N* = 83)	.973 [.965, .981] (.037, *N* = 82)	2.99 [2.63, 3.34] (1.65, *N* = 85)	2.31 [2.01, 2.61] (1.41, *N* = 85)	2.78 [2.59, 2.96] (.86, *N* = 85)
Bisexual (Kinsey 2–4)	.40 [.16, .62]* (.73, *N* = 42)	.20 [.02, .38] (.60, *N* = 46)	.990 [.977, 1.00]* (.040, *N* = 38)	.990 [.974, 1.00]* (.047, *N* = 38)	3.63 [3.17, 4.10]* (1.81, *N* = 45)	2.73 [2.35, 3.11] (1.26, *N* = 45)	3.11 [2.86, 3.36]^†^ (.81, *N* = 43)
Homosexual (Kinsey 5–6)	.33 [.11, .55]* (.84, *N* = 60)	.36 [.18, .53]^†^ (.71, *N* = 65)	.967 [.959, .974] (.029, *N* = 62)	.970 [.961, .979] (.035, *N* = 62)	3.87 [3.42, 4.31]* (1.80, *N* = 66)	3.41 [2.98, 3.84]* (1.75, *N* = 66)	3.67 [3.34, 4.00]* (1.30, *N* = 63)

Numbers in square brackets represent 95% confidence intervals of the mean. Numbers in parentheses represent standard deviations of the mean and sample sizes. Participants were grouped according to their scores on the Kinsey scale, with Kinsey 0–1 considered heterosexual, 2–4 considered bisexual, and 5–6 considered homosexual. Asterisks indicate significant difference to heterosexual, ^†^*p* < .10, *p < .05

### Measures and Materials

#### Self-Reported Sexual Orientation

Participants reported both their sexual orientation identity and sexual attraction to men and women on 7-point scales (Kinsey et al., 1953). These scales were highly correlated, *p* < .0001, *r* = 0.97, 95% CI [0.95, 0.97], and averaged within participants. For this average, a score of 0 represented exclusive heterosexuality, a score of 3 bisexuality with equal attractions to women and men, and 6 represented exclusive homosexuality. This composite score was used for all analyses. Note that the sexual orientation variable treated as continuous variable for all analyses, with the exception of the list of descriptive statistics found in Table 1.

#### Self-Reported Masculinity–Femininity

Childhood masculinity was assessed using six items from the Childhood Gender Nonconformity Scale, and adulthood behavioral masculinity was measured using six items from the Continuous Gender Identity Scale. These scales produce sexual orientation differences in masculinity–femininity in the predicted directions (Rieger et al., 2008, 2010). However, some of the items used outdated wording or were targeted toward an U.S. (rather than UK) sample. We therefore removed one item of the original childhood scale and completely reworded items of the adulthood scale (see Appendix for a full list of items). Participants responded to statements on 7-point scales ranging from 1 (strongly disagree) to 7 (strongly agree). Answers were scored such that higher numbers represented greater masculinity. Item reliability (Cronbach’s alpha) was 0.89 for the Childhood Gender Nonconformity Scale and 0.93 for the Continuous Gender Identity Scale. Because the adulthood scale was entirely reworded, we conducted further checks on its psychometric properties. It correlated with sexual orientation (*r* = 0.36), self-report from childhood (*r* = 0.67), and observer ratings from adulthood (*r* = 0.48) in the same manner, if not slightly better, than the corresponding correlations across heterosexual and homosexual women in research using the previous version (Rieger et al, 2008, 2010), lending support to its convergent validity.

#### 2D:4D

Digit measurements were taken from either high-resolution photographs or scans of participants’ hands, similar to past work (Allaway et al., 2009; Watts et al., 2018a). For the photographs, participants placed their hands on a flat surface with their fingers slightly spread apart, and images were taken from approximately 30 cm above this surface. For the scans, participants placed their hands flat on the surface of the scanner. Different methods of capturing images (photograph or scanner) did not moderate the relationship between sexual orientation and 2D:4D.

From the resulting images of hands, digit ratios were measured by two independent raters who were blind to the sex and sexual orientation of the participants. Raters used the open-source vector graphics package Inkscape 0.92, as computer-assisted measurement techniques produce highly reliable measurements (Watts et al., 2018a). Each rater drew a line as wide as the finger along the lowest crease at the base of the finger, between the metacarpal and proximal phalanx. A second line was then drawn from the tip of the finger down toward the base, where it automatically snapped to the center of the base line. Fine adjustments were then made at a higher level of zoom, to ensure that this line matched the tip of the finger as closely as possible. Inter-rater reliability (Cronbach’s alpha) exceeded 0.99 for each digit. Therefore, the measurements for each digit were averaged between raters. Finally, 2D:4D was calculated by dividing the averaged length of the index finger by the averaged length of the ring finger for each hand of each participant.

#### Stimuli

The sexual stimuli consisted of 3-min videos, three featuring a female model and three featuring a male model, each of them masturbating in a bedroom. These stimuli were selected in a previous study in which 200 videos were rated on their sexual appeal by men and women of different sexual orientations (Rieger et al., 2015), and the top three female and male videos were used in the present study. Neutral stimuli to assess baseline genital responses were 2-min clips taken from a nature documentary. Their engaging but non-sexual content facilitated participants’ return to an unaroused level. However, these nature videos were not used for pupil dilation baseline, as their engaging content might elicit dilation for reasons other than sexual arousal. Thus, two 1-min animations of clouds were used to obtain a pupillary baseline. All videos were edited using MPEG Streamclip and Final Cut Pro to be of similar luminance.

#### Genital Arousal

Genital arousal was measured as changes in peak-to-trough vaginal pulse amplitude (VPA) using a vaginal photoplethysmograph. The signal was recorded using a BIOPAC MP150 data acquisition unit, sampled at 200 Hz, and high-pass-filtered at 0.5 Hz with 16-bit resolution. The VPA exhibits both convergent and discriminant validity for the measurement of female sexual response (Suschinsky et al., 2009).

#### Pupil Dilation

Pupil dilation data were measured with a SR Research EyeLink 1000 infrared eye tracking unit. A 35 mm lens focused on the participants’ right eye, positioned approximately 60 cm from the participants’ head, and sampling at a rate of 500 Hz. The infrared light emitted by the eye tracker is reflected by the pupil, and the number of pixels reflected were recorded. Because raw pupil area data included “0’s” for missing values, for instance from blinks or head movements, these values were removed prior to analyses.

### Procedure

#### Participant Session

After giving written informed consent, participants completed a survey on their demographics, sexual orientation, and masculinity–femininity, and had photographs or scans of their hands taken. They were then seated in a chair and had their entire body video-recorded for 5–10 min to capture their gestures and movements. Participants answered questions about the weather and their interests and were not interrupted while answering, nor were participants informed that these videos would be used to assess their masculinity–femininity, but rather were told that they would be rated for “measures of psychological interest.” For our observer ratings of masculinity–femininity we used their answer to a neutral question: “How would you describe the weather at this time of year?”

Participants were then seated in a sealed booth, with dim lighting conditions. Eyes were calibrated by participants fixating on dots outlining the screen. They were then instructed on how to use the genital measure, which they inserted in privacy after the experimenter left the booth (and at which point both their eye data and genital data were checked remotely). Pupil dilation and genital arousal were measured simultaneously throughout the experiment. Participants were instructed to watch the screen throughout the experiment, regardless of the content. They first viewed an animation of clouds, followed by alternating sexual and nature videos. These were displayed in a random order, but a sexual video was always followed by a nature video. After each nature video, the experiment displayed a gray screen of similar luminance to the videos until the experimenter verified that participants had returned to baseline arousal for a minimum of 5 s. Following this, the next erotic video was shown. After the sixth nature video, a final animation of clouds was displayed. Participants were paid £50. The entire procedure took approximately 90 min.

For each participant, genital data and pupil data were averaged across the duration of each stimulus. These averages were then standardized within participants, producing a z-score for each participant and stimulus. For genital data, standardized responses to the 5 s preceding each sexual stimulus (following the display of a neutral stimulus, and after the participant had returned to baseline) were subtracted from the standardized response to the sexual stimulus. For pupil data, standardized responses to neutral stimuli (the animated clouds) were subtracted from standardized responses to all sexual stimuli. We then computed, for each participant, average responses across all sexual stimuli of a given type (female or male), which reflected their responses to each sex as compared to baseline. These scores were then used to calculate a contrast score representing their response to females over males, such that a positive score indicated a preference for females, a negative score indicated a preference for males, and a score of zero indicated equal preferences. Caution must be taken when producing such a contrast score from genital arousal scores of heterosexual women, because this can lead to the averaging of responses which are individually very different, which produces the illusion of a non-specific response on a group level (Lalumière et al., 2020). We therefore checked whether this was the case in the present sample. The distribution of genital scores for heterosexual women were normally distributed and centered on zero, and their mean was not significantly different from zero. As such, we are confident that averaging the genital arousal scores of heterosexual women does not distort the pattern of the data in the present sample.

#### Editing of Participant Videos

Recordings of participants’ answers to our question about the weather were edited in Shotcut and used for analyses. We selected the first sentence that the participants articulated within the first 20 s of their answer. If responses were less than 6 s, we took a combination of their first and second sentence. The majority of selected videos were approximately 10 s long, and all clips included audio. Raters can reliably judge behavioral traits associated with sexual orientation from brief video clips such as these (Tskhay & Rule, 2013).

#### Ratings of Masculinity–Femininity

Psychology students participated as raters of masculinity–femininity for course credit, and each video-recorded target was evaluated by a minimum of 21 and a maximum of 46 raters. In total, we had 48 heterosexual male raters, 21 non-heterosexual male raters, 71 heterosexual female raters, and 29 non-heterosexual female raters. Videos were rated in batches of 20–30 to avoid rater fatigue, and raters from each rater group were randomly assigned to a batch.

Raters were blind to the participants’ sexual orientation. They were not trained in how to rate, but instructed to indicate their impression of each woman’s appearance and demeanor, in comparison with other women of the same age. For example, they were told to “rate whether this woman appeared or behaved in a more feminine or masculine way.” Ratings were completed on 7-point scales, where a score of 1 was “more feminine,” 4 “average,” and 7 “more masculine.” Heterosexual raters tended to give higher scores than non-heterosexual raters [mean (SD): heterosexual males: 3.05 (1.04); heterosexual females: 3.29 (1.17); non-heterosexual males: 2.59 (1.09); non-heterosexual females: 2.89 (1.26)], but correlations between rater groups (heterosexual and non-heterosexual men and women) ranged from *r* = 0.68 to *r* = 0.81, and all relationships were positive. Additionally, ratings were highly reliable within each rater group and across all raters (all Cronbach’s α’s > 0.95). Evaluations were therefore averaged across all raters, producing an average observer-rated masculinity–femininity score for each video-recorded participant.

## Results

### Initial Analyses

Although we treat sexual orientation as a continuous variable in all analyses, we first present a summary of our key variables with participants grouped according to their scores on the Kinsey scale, with Kinsey 0–1 considered heterosexual, 2–4 considered bisexual, and 5–6 considered homosexual (Table 1). Significance values are also given, using heterosexuals as the comparison group. Unexpectedly, bisexual women as a group had more feminine 2D:4D ratios than both heterosexual and homosexual women. Also note that on average, heterosexual and homosexual women did not differ in their digit ratios.

#### Hypothesis 1

We hypothesized that homosexual women would be more sexually aroused to stimuli featuring females, whereas heterosexual women would show similar arousal to both sexes, and that bisexual women would be intermediate between the groups. We regressed women’s responses to sexual stimuli onto their sexual orientation. For each measure of sexual arousal (genital arousal or pupil dilation), we had three dependent variables: their responses to females, their responses to males, and their responses to females over males. We originally tested for both a linear and curvilinear effect of sexual orientation on women’s sexual responses, to account for the possibility that differences between heterosexual, bisexual, and homosexual women may not always follow a simple linear trend (Rieger et al., 2016). However, in the present data, the curvilinear effects did not explain arousal over and above linear effects, further suggesting that bisexual women were intermediate between heterosexual and homosexual women in the below effects. For the sake of simplicity, in the following we focus on analyses in which only linear effects of sexual orientation were tested.

**Fig. 1:**
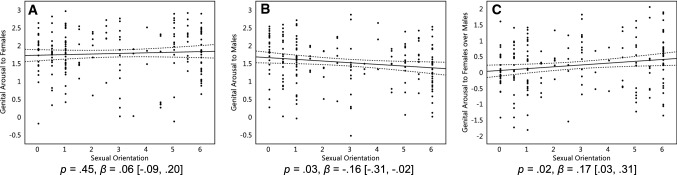
Women’s genital responses to sexual stimuli. Genital arousal of 184 women in response to stimuli featuring females (**a**), males (**b**), and females over males (**c**). On the Y axis, genital arousal scores reflect changes compared to the 5 s preceding a sexual stimulus, standardized within participants. On the X axis, 0 represents exclusive heterosexuality, 3 bisexuality, and 6 represents exclusive homosexuality. Triple lines represent regression coefficients with their 95% confidence intervals. Dots represent participants’ average scores

We first regressed women’s genital arousal to female stimuli and male stimuli onto their sexual orientation, which was treated as a continuum. Given the nonsignificant linear effect of sexual orientation, homosexual women did not respond significantly more to females as compared with heterosexual and bisexual women, *p* = .45, *β* = 0.06, 95% CI [− 0.09, 0.20] (Fig. 1a). Homosexual women did respond significantly less to males than did other women, *p* = .03, *β* = − 0.16 [− 0.31, − 0.02] (Fig. 1b). We then regressed women’s genital arousal contrast of females over males onto their sexual orientation. Homosexual women responded significantly more to their preferred sex (females) than their non-preferred sex (males) as compared with heterosexual women, *p* = .02, *β* = 0.17 [0.03, 0.31], and bisexual women were intermediate between the other groups (Fig. 1c).

Independent of these patterns, women, in general, responded significantly to both females and males as compared to baseline. That is, across sexual orientations, the confidence intervals of the regression coefficients were above 0 (Fig. 1a, b).

**Fig. 2:**
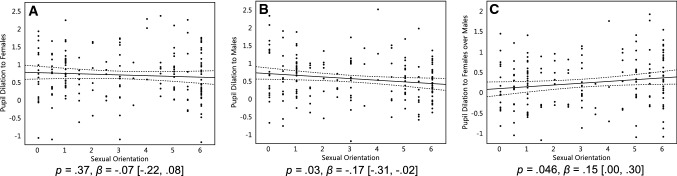
Women’s pupil dilation to sexual stimuli. Pupil dilation of 175 women in response to stimuli featuring females (**a**), males (**b**), and females over males (**c**). On the Y axis, pupil dilation scores reflect changes compared to the neutral stimuli, standardized within participants. On the X axis, 0 represents exclusive heterosexuality, 3 represents bisexuality, and 6 represents exclusive homosexuality. Triple lines represent regression coefficients with their 95% confidence intervals. Dots represent participants’ average scores

We then repeated the above analyses with pupil dilation to sexual stimuli as the dependent variable. Results were similar to those for genital arousal. Homosexual women did not respond significantly more to females as compared with heterosexual and bisexual women, *p* = .37, *β* = − 0.07 [− 0.22, 0.08] (Fig. 2a), but responded significantly less to males as compared with other women, *p* = .03, *β* = − 0.17 [− 0.31, − 0.02] (Fig. 2b). We then regressed women’s pupil dilation to females over males onto their sexual orientation. Homosexual women responded significantly more to females than males as compared with heterosexual women, *p* = .046, *β* = 0.15 [0.00, 0.30], and again, bisexual women were intermediate (Fig. 2c). As with genital arousal, women of all sexual orientations responded significantly to both female and male sexual stimuli as compared to baseline (i.e., the confidence intervals of the effects were above 0; Fig. 2a, b).

#### Hypothesis 2

**Fig. 3:**
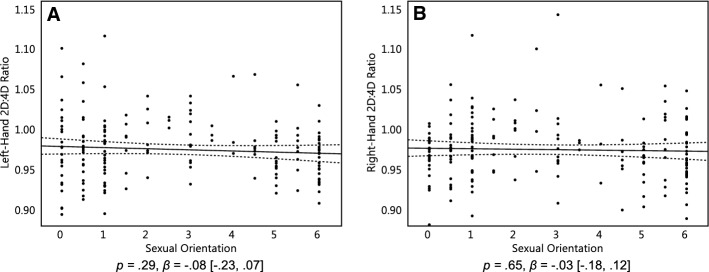
Left-hand 2D:4D of 183 women (**a**) and the right-hand 2D:4D of 182 women (**b**). On the Y axis, 2D:4D is the length of the index finger divided by the length of the ring finger. On the X axis, 0 represents exclusive heterosexuality, 3 represents bisexuality, and 6 represents exclusive homosexuality. Triple lines represent regression coefficients with their 95% confidence intervals. Dots represent participants’ scores

We hypothesized that homosexual women have more male-typical (lower) 2D:4D than heterosexual women, and that bisexual women would be intermediate between the two. We regressed women’s left-hand and right-hand 2D:4D onto their sexual orientation. We found no significant linear relationship between women's sexual orientation and their 2D:4D in either their left hand, *p* = .29, *β* = − 0.08 [− 0.23, 0.07], or their right hand, *p* = .65, *β* = − 0.03 [− 0.18, 0.12] (Fig. 3a, b).

As aforementioned, Table 1 shows that bisexual women had, unexpectedly, higher 2D:4D than both heterosexual and homosexual women. We therefore tested whether the negative quadratic effect of sexual orientation on 2D:4D was significant (i.e., bisexual women had more feminine 2D:4D than both heterosexual and homosexual women), and found that it was significant in both the left hand, *p* = .02, *β* = − 0.19 [− 0.35, − 0.02] and the right hand, *p* = .003, *β* = − 0.25 [− 0.41, − 0.09]. However, even when excluding bisexual women, homosexual women did not have more male-typical ratios than heterosexual women (this can also be seen in Table 1), plus the pattern of subsequent mediation analyses remained identical, with or without bisexual women excluded. All further reported results are therefore from analyses in which bisexual women were included.

**Fig. 4:**
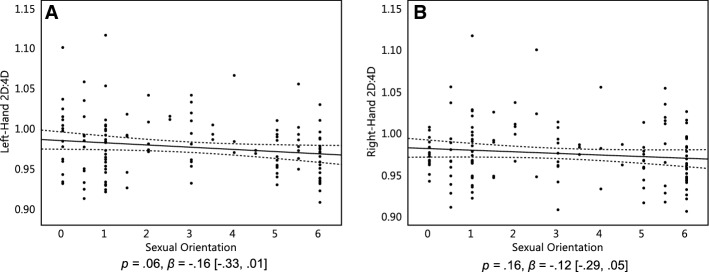
Left-hand 2D:4D of 136 Caucasian women (**a**) and the right-hand 2D:4D of 135 Caucasian women (**b**). On the Y axis, 2D:4D is the length of the index finger divided by the length of the ring finger. On the X axis, 0 represents exclusive heterosexuality, 3 represents bisexuality, and 6 represents exclusive homosexuality. Triple lines represent regression coefficients with their 95% confidence intervals. Dots represent participants’ scores

Furthermore, there is evidence from one study that ethnicity can influence the relationship of women's sexual orientation with their 2D:4D (Lippa, 2003), even though there is no evidence for this from a meta-analysis (Grimbos et al., 2010). Still, we repeated the analysis for only the 136 Caucasian participants, as they formed the majority of our sample. Despite the reduced sample size, the linear relationship between 2D:4D and sexual orientation was closer to significance in both the left hand, *p* = .06, *β* = − 0.16 [− 0.33, 0.01], and their right hand, *p* = .16, *β* = − 0.12 [− 0.29, 0.05] (Fig. 4a, b). However, the inclusion or exclusion of nonwhite ethnicities did not affect the patterns of subsequent analyses mediation analyses, and we therefore kept nonwhite ethnicities included.

#### Hypothesis 3

We hypothesized that the relationship between sexual orientation and male-typical sexual arousal in women is mediated by their male-typical 2D:4D. Although the linear relationship between 2D:4D and sexual orientation was not significant, we conducted this analysis regardless, as it was planned in advance. We computed multiple regression analyses predicting the genital arousal or pupil dilation contrast (responses to females over males) by sexual orientation and digit ratios. We focused on the contrast score since it tended to show, across the two measures of sexual response, a somewhat stronger relation with sexual orientation than responses to females or responses to males (Figs. 1and2).

In Step 1, sexual orientation was the only predictor of sexual response. In Step 2, we included a 2D:4D variable—either left or right hand—as a mediator. If our hypothesis were confirmed, then the inclusion of 2D:4D as a mediator should weaken the relationship between sexual orientation and either measure of sexual arousal. Tables 2 and 3 summarize the results of the analyses both for genital arousal and for pupil dilation, for Step 1 (without 2D:4D as mediator) and Step 2 (with 2D:4D as mediator). The effect of sexual orientation on sexual arousal remained similar (or slightly increased) after including 2D:4D as a predictor. Furthermore, a mediation analysis on the basis of 10,000 bootstrapped samples (Preacher & Hayes, 2008) did not indicate that 2D:4D mediated the relationship between sexual orientation and sexual responses, because the confidence intervals of the estimated mediation effects included zero. This was the case for left-hand 2D:4D predicting genital arousal, *β* = -0.01 [− 0.04, 0.01], and pupil dilation *β* = 0.007 [− 0.01, 0.04]. It was also the case for right-hand 2D:4D predicting genital arousal *β* = − 0.005 [− 0.03, 0.02], and pupil dilation *β* = − 0.003 [− 0.02, 0.02].

**Table 2:** Multiple regression analyses for sexual orientation and left-hand 2D:4D predicting genital arousal (Step 1 *N* = 184, Step 2 *N* = 174) and pupil dilation (Step 1 *N* = 175, Step 2 *N* = 160) to females over males

Step 1	Genital arousal to females over males	Pupil dilation to females over males
Variables	*β*	*β*
Sexual orientation (SO)^1^	.17 [.03, .31]*	.15 [.00, .30]*
Step 2	Genital arousal to females over males	Pupil dilation to females over males
Variables	*β*	*Β*
Sexual orientation (SO)^1^	.20 [.05, .35]**	.14 [− .02, .30]^†^
Left-hand 2D:4D^2^	.13 [− .02, .28]^†^	− .05 [− .21, .11]

*R*^2^’*s* for the two models are .03 and .02 in Step 1, and .05 and .02 in Step 2. Numbers in brackets represent 95% confidence intervals of the standardized regression coefficient, *β*. ^1^Higher scores indicate a more homosexual orientation. ^2^Lower scores indicate more male-typical 2D:4D. ^†^*p* < .10, *p < .05, **p < .01

**Table 3:** Multiple regression analyses for sexual orientation and right-hand 2D:4D predicting genital arousal (Step 1 *N* = 184, Step 2 *N* = 173) and pupil dilation (Step 1 *N* = 175, Step 2 *N* = 160) to females over males

	Genital arousal to females over males	Pupil dilation to females over males
*Step 1*		
Variables	*β*	*Β*
Sexual orientation (SO)^a^	.17 [.03, .31]*	.15 [.00, .30]*
*Step 2*		
Variables	*β*	*β*
Sexual orientation (SO)^a^	.19 [.04, .33]*	.15 [.00, .31]^†^
Right-hand 2D:4D^b^	.15 [.01, .30]*	.03 [-.13, .19]

*R*^2^’*s* for the two models are .03 and .02 in Step 1, and .06 and .02 in Step 2. Numbers in brackets represent 95% confidence intervals of the standardized regression coefficient, *β*. ^a^Higher scores indicate a more homosexual orientation. ^b^Lower scores indicate more male-typical 2D:4D. ^†^*p* < .10, *p < .05, **p < .01

#### Hypothesis 4

**Fig. 5:**
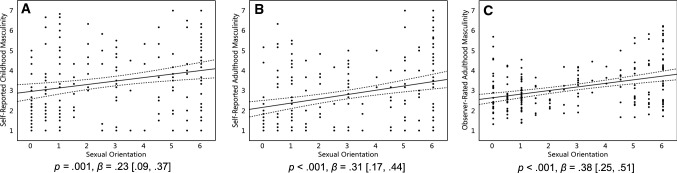
Childhood and adulthood self-report and observer-rated behavioral masculinity data of 192 women (self-report) and 191 women (observer ratings). On the Y axis, behavioral masculinity scores, with higher scores representing a greater degree of masculinity. On the X axis, 0 represents exclusive heterosexuality, 3 represents bisexuality, and 6 represents exclusive homosexuality. Triple lines represent regression coefficients with their 95% confidence intervals. Dots represent participants’ scores

We hypothesized that homosexual women were more masculine and less feminine in their self-report and behaviors than heterosexual women, and that bisexual women would be intermediate between the two groups. We regressed women’s self-reported adulthood and childhood masculinity–femininity, in addition to observer ratings of their masculinity–femininity, onto their sexual orientation. As for sexual response, there were no significant quadratic effects of sexual orientation on masculinity–femininity, and we therefore focused on linear effects. Homosexual women were significantly more masculine than heterosexual women in their self-reports of childhood, *p* = .001, *β* = 0.23 [0.09, 0.37], and adulthood, *p* < .001, *β* = 0.31 [0.17, 0.44], and when rated by others, *p* < .001, *β* = 0.38 [0.25, 0.51] (Fig. 5a–c). For all three measures of gender nonconformity, bisexual women were intermediate between heterosexual and homosexual women (Table 1).

#### Hypothesis 5

We hypothesized that the effect of sexual orientation on male-typical sexual arousal was mediated by women’s higher levels of masculinity. To investigate this, we computed a total of 6 sets of regression analyses with 2 steps each, predicting either genital arousal or pupil dilation to females over males. In each analysis, in Step 1, sexual orientation was the only predictor of sexual response. In Step 2, one measure of masculinity–femininity was included alongside sexual orientation as a predictor: either self-report from childhood or adulthood, or observer ratings from adulthood. If male-typical sexual responses in homosexual women were mediated by their masculinity, then the inclusion of a measure of masculinity should weaken the relationship of sexual orientation with sexual arousal.

Tables 4, 5, and 6 summarize the results of these analyses. In general, sexual orientation effects on sexual response did not decrease (and if anything, increased) with a measure of masculinity–femininity as a covariate. Likewise, mediation analyses on the basis of 10,000 bootstrapped samples (Preacher & Hayes, 2008) did not suggest mediation by any measure of masculinity on either measure of sexual response.

**Table 4:** Multiple regression analyses for sexual orientation and self-reported childhood masculinity predicting genital arousal (Step 1 *N* = 184, Step 2 *N* = 181) and pupil dilation (Step 1 *N* = 175, Step 2 *N* = 172) to females over males

	Genital arousal to females over males	Pupil dilation to females over males
*Step 1*		
Variables	*β*	*β*
Sexual orientation (SO)^a^	.17 [.03, .31]*	.15 [.00, .30]*
*Step 2*		
Variables	*β*	*Β*
Sexual orientation (SO)^a^	.18 [.03, .33]*	.18 [.02, .33]*
Self-reported childhood masculinity^b^	.00 [-.15, .15]	− .11 [− .26, .05]

*R*^2^’*s* for the two models are .03 and .02 in Step 1, and .03 and .03 in Step 2. Numbers in brackets represent 95% confidence intervals of the standardized regression coefficient, *β*. ^a^Higher scores indicate a more homosexual orientation. ^b^Higher scores indicate higher self-reported childhood behavioral masculinity. ^†^*p* < .10, *p < .05, **p < .01

**Table 5:** Multiple regression analyses for sexual orientation and self-reported adulthood masculinity predicting genital arousal (Step 1 *N* = 184, Step 2 *N* = 181) and pupil dilation (Step 1 *N* = 175, Step 2 *N* = 172) to females over males

	Genital arousal to females over males	Pupil dilation to females over males
*Step 1*		
Variables	*β*	*β*
Sexual orientation (SO)^a^	.17 [.03, .31]*	.15 [.00, .30]*
*Step 2*		
Variables	*β*	*β*
Sexual orientation (SO)^a^	.19 [.04, .35]*	.19 [.03, .35]*
Self-reported adulthood masculinity^b^	− .05 [-.20, .10]	− .12 [− .28, .04]

*R*^2^’*s* for the two models are .03 and .02 in Step 1, and .03 and .03 in Step 2. Numbers in brackets represent 95% confidence intervals of the standardized regression coefficient, *β*. ^a^Higher scores indicate a more homosexual orientation. ^b^Higher scores indicate higher self-reported adulthood behavioral masculinity. ^†^*p* < .10, *p < .05, **p < .01

**Table 6:** Multiple regression analyses for sexual orientation and video observer-rated adulthood masculinity predicting genital arousal (Step 1 *N* = 184, Step 2 *N* = 180) and Pupil Dilation (Step 1 *N* = 175, Step 2 *N* = 167) to females over males

	Genital arousal to females over males	Pupil dilation to females over males
*Step 1*		
Variables	*β*	*β*
Sexual orientation (SO)^a^	.17 [.03, .31]*	.15 [.00, .30]*
*Step 2*		
Variables	*β*	*β*
Sexual orientation (SO)^a^	.17 [.01, .33]*	.15 [-.02, .31]
Observer-rated masculinity^b^	− .04 [− .19, .12]	− .01 [− .17, .16]

*R*^*2*^*’s* for the two models are .03 and .02 in Step 1, and .03 and .02 in Step 2. Numbers in brackets represent 95% confidence intervals of the standardized regression coefficient, *β*. ^a^Higher scores indicate a more homosexual orientation. ^b^Higher scores indicate higher observer-rated masculinity. ^†^*p* < .10, *p < .05, **p < .01

## Discussion

The present data confirmed that homosexual women had more male-typical sexual arousal patterns than heterosexual women, as indicated by both their genital arousal and their pupil dilation. However, there was no evidence that they had more male-typical digit ratios, or that digit ratios mediated the relationship between women’s sexual orientation and their male-typical sexual arousal patterns. Moreover, even though homosexual women were more masculine than heterosexual women in their self-reports or via observer ratings, this pattern, too, did not explain their male-typical arousal patterns.

The finding that homosexual women had stronger responses to their preferred sex than heterosexual women is consistent with previous research both for genital arousal and pupil dilation (Chivers et al., 2004, 2007; Rieger et al., 2015, 2016). However, the finding that 2D:4D was not significantly lower in homosexual women than heterosexual women is puzzling, as it was confirmed previously in a meta-analysis (Grimbos et al., 2010). This may have been due to methodological reasons: Although between-rater reliability was high, and computer-assisted measurement techniques, such as those employed in the current study, have been shown to have the highest reliability compared to other methods of measuring 2D:4D (Allaway et al., 2009), we cannot say with certainty that our measure was valid.

Indeed, there is an ongoing debate about the utility of 2D:4D: Although it is regarded as a valid measure with respect to sex differences and female sexual orientation differences, it is also the case that there is much variability in this measure across individuals, and findings only apply on aggregate and do not apply to single people (Swift-Gallant et al., 2020). Furthermore, the aforementioned meta-analysis suggested a publication bias in reported relationships of sexual orientation with 2D:4D (Grimbos et al., 2010), and the true effect could therefore be smaller than usually published. In the present data, the strongest linear relationship of sexual orientation with 2D:4D was *r* (or *β*) = − 0.12 in the right hand. With this effect, post hoc power analyses indicated a minimum sample of 542 women for it to be significant. If our a priori sample size estimate had returned such a large number, we would have considered it an unreasonable goal for a laboratory-based study like ours.

Another possible explanation for the present null finding with respect to 2D:4D is the ethnic makeup of the sample. We did not factor this into planning the present study because the meta-analysis pointed to an ethnicity effect only in men and not in women (Grimbos et al., 2010), although other research has found an influence of ethnicity on 2D:4D in women (Lippa, 2003). Indeed, excluding all non-Caucasian participants from the present sample made the association between 2D:4D and sexual orientation stronger (although still nonsignificant) in both hands (Fig. 4). Thus, future research measuring the relationship between 2D:4D and sexual orientation may wish to either employ a racially homogenous participant sample, or recruit enough participants that per-race comparisons are feasible. Note that even within the white sample, 2D:4D did not appear to explain (mediate) any relationship of women's sexual orientation with their sexual response patterns.

It is impossible to draw any conclusions from the present data about whether the relationship between 2D:4D and sexual orientation mediates the relationship between sexual orientation and sexual responses, simply because 2D:4D in itself did not relate to sexual orientation. With regard to masculinity–femininity, if anything, statistically controlling for any of the three masculinity–femininity variables made the correspondence of women’s sexual orientation with their male-typical sexual arousal stronger. This pattern—a strengthening of the effect of sexual orientation on sexual response when measures of behavioral masculinity are statistically controlled for—has been previously noted (Rieger et al., 2016). In combination with present findings, it appears unlikely that it was previously a chance finding.

If one assumed for a moment that the present findings are accurate, what could be their reasons? For females, it is possible that there exist several “sensitive periods” of masculinization during prenatal development, and that these periods differ for different traits (McCarthy et al., 2018; Xu et al., 2019). At least in non-human primates, exposure to testosterone at different stages of gestation may masculinize sexual behaviors independently from non-sexual behaviors (Goy et al., 1988). Specifically, Goy et al. reported that female rhesus macaques exposed to testosterone during their prenatal development had different behavioral outcomes depending on the timing, with those exposed early in gestation displaying male-typical sexual behaviors (e.g., mounting other females) and those exposed late in gestation displaying male-typical non-sexual behaviors (e.g., rough play). It is possible that behavioral traits and sexual arousal patterns are masculinized at different stages of development in humans also, and thus, are not necessarily interlinked within individuals—for example, those who have male-typical arousal may not have male-typical gender-related behaviors and vice versa.

A final point concerns bisexual women, who were intermediate between heterosexual and homosexual women in their sexual arousal and masculinity–femininity, but were significantly more feminine in their 2D:4D. One hypothesis is that due to intermediate dosages of genetic or prenatal hormonal influences, bisexual individuals, who could be considered to have sexual orientations between heterosexual and homosexual, also fall intermediate with respect to correlates of sexual orientation (Rieger et al., 2020). Thus, regarding bisexual women's 2D:4D, we assumed that they could also be intermediate between heterosexual and homosexual women on this measure. Contrary to this assumption, bisexual women had more feminine 2D:4D than both heterosexual and homosexual women (Table 1). It has been proposed that personality differences between homosexual and heterosexual women may be caused by exposure to androgens during prenatal development, whereas the distinct personality traits of bisexual individuals (e.g., higher sociosexuality compared to heterosexual and homosexual) may be a correlate of their higher levels of postnatal androgens (Lippa, 2020). If the present findings are valid, they would suggest that bisexual women also differ from heterosexual and homosexual women with respect to prenatal androgenization, but this would imply that they have been less masculinized than other groups, and we cannot offer an explanation for why this would be the case.

In sum, the findings of the present research suggest that there is no link between the male-typical sexual responses of homosexual women and putative markers of prenatal androgenization. Other purported markers of androgen exposure may reveal a different pattern than the one reported here. Such markers include the distance between the anus and the genitalia (Barrett et al., 2018) and otoacoustic emissions, which are tiny sounds emitted by the inner ear (McFadden & Pasanen, 1998). Another avenue for future research would involve individuals with conditions affecting the availability of androgens, or their sensitivity to them. To our knowledge, no studies to date investigated the arousal patterns of women with CAH. If androgen exposure does indeed impact sexual responses—and given the apparent impact of excessive androgens on the sexual orientation of women with CAH (Meyer-Bahlburg et al., 2008; Zucker et al., 1996)—women with CAH may show male-typical specificity in their sexual arousal.

## Appendix

Scale Items: Childhood Gender Nonconformity Scale (Women).

As a child, I felt that I had more in common with boys than girls.

As a child, I was perceived as masculine by my peers.

As a child, I preferred playing with boys rather than girls.

I was more masculine than feminine.

As a child, I sometimes wished I had been born a boy rather than a girl.

As a child, I usually avoided feminine clothing (such as dresses).

Scale Items: Adulthood Continuous Gender Identity Scale (Women).

My mannerisms are not very feminine.

I assume most people see me as not very feminine.

I assume most people see me as masculine.

I consider myself very masculine in my behaviors and interests.

I do not consider myself very feminine in my behaviors and interests.

I consider myself more masculine than feminine.
